# Comparative effects of different types and doses of biochar on soil quality indicators and arugula growth under saline conditions

**DOI:** 10.1038/s41598-025-92816-w

**Published:** 2025-03-24

**Authors:** Naglaa Khalaf ELsaman, Abu El-Eyuoon Abu Zied Amin, Mohamed Abd El-Razek, Nadia Mohamed Kamal Roshdy

**Affiliations:** Soils and Water Department, Faculty of Agriculture, Assiut University, P.O. Box: 71526, Assiut, Egypt

**Keywords:** Arugula, Biochar, Saline soil, Saline water, Sustainable agriculture, Plant sciences, Environmental sciences

## Abstract

Population and food demand increased rapidly so to face this increment; we must dramatically increase food crop production to ensure global food security. Hence, saline agriculture is a possible solution for producing food in salt-affected soils using saline water for irrigation. The objectives of this study were to investigate the effects of applying different types and doses of biochar to saline soil under irrigation by saline water on soil quality indicators and growth parameters and yield of arugula plant. Four types of biochar: banana leaves biochar (BLB), rice straw biochar (RSB), sorghum stalks biochar (SSB), and wood chips biochar (WCB) were applied to the soil in the pots at levels of 1%, 3%, and 5% (w/w). This pot experiment was cultivated by arugula under irrigation with saline water (6.2 dS m^− 1^). Total available nitrogen increased significantly relative to the control treatment (unamended soil) by 41%, 34%, 43%, 34%, 33%, 24%, 41%, and 44% under adding 3%WCB, 5%WCB, 1%BLB, 3%BLB, 5%BLB, 5%SSB, 1%RSB, and 5%RSB treatments, respectively. Results showed significant increases in available potassium (K) over the control treatment by 48%, 125%, 410%, 738%, 137%, 352%, 632%, 158%, 576%, and 849% for 5%WCB, 1%BLB, 3%BLB, 5%BLB, 1%SSB, 3%SSB, 5%SSB, 1%RSB, 3%RSB, and 5%RSB treatments, respectively. Cation exchange capacity increased significantly relative to the control treatment by 26%, 22%, 30%, 58%, 31%, 54%, 28%, and 48% for 3%WCB, 5%WCB, 3%BLB, 5%BLB, 3%SSB, 5%SSB, 3%RSB, and 5%RSB, respectively. Relative to the control treatment, the fresh biomass of the arugula plant significantly improved by 97%, 143%, 76%, 129%, 103%, 146%, 81%, 57%, 121%, and 97% for 3%WCB, 5%WCB, 1%BLB, 3%BLB, 1%SSB, 3%SSB, 5%SSB, 1%RSB, 3%RSB, and 5%RSB, respectively. The highest value of fresh biomass, nitrogen uptake, and phosphorus uptake of arugula plant were observed at 3%SSB applications. According to the results obtained from our study, we recommend adding sorghum stalks biochar at 3% which is a promising approach to rehabilitate saline soil and use saline water for sustainable crop production, this is attributed to the effective improvement of the nutrient uptake, productivity, and growth of arugula plant under saline conditions as it enhances the tolerance of plants under salt stress as well as improved nutrient supply and soil quality. Also, adding 3% sorghum stalks biochar saves the costs of addition and production compared to adding 5% dose. This study also provided useful information about the optimal quantities and types of biochar used to improve the productivity of saline soils.

## Introduction

Global climate changes affect weather patterns, affecting soil salinity and its ability to withstand drought^1^. Arid and semi-arid regions worldwide are frequently threatened by water shortage and soil salinization, which deteriorate soil quality indicators, restrict sustainable agricultural development, and cause serious damage to the ecosystems^2,3^. Soil salinity occurs as a result of many natural and anthropogenic factors^1^. Hence, managing salt-affected soils is an important strategy in cultivating these soils^4^. The productivity of saline soils can be maximized by cultivating salt-tolerant crops to meet global food demand^5^. The presence of salts in the soil, prevents crops from absorbing nutrients, resulting in nutritional disorders^6^. Several strategies have been applied to reclaim salt-affected soils, such as hydraulic, chemical, and phytoremediation techniques^7^. Water is an essential component of agriculture and a key social and economic driver of sustainable growth, livelihoods, justice, food security, and employment. The agriculture sector consumes approximately 70% of the total global freshwater used^8^. Rapid population growth led to a continuous decline in freshwater supplies for agriculture. In regions suffering from a shortage of fresh water, saline water has been confirmed to be an alternative source of irrigation for agriculture^9^. Nowadays, using non-conventional water sources in agriculture has become necessary to cope with the increasing population around the world^10^.

Converting crop residues into biochar used as an amendment is one of the most promising strategies in sustainable agriculture^11^. Feedstock type plays a major role in affecting the properties of biochar produced at low temperatures and its suitability for application in arid regions^12^. Biochar applications to the soils decreased bulk density, and enhanced aggregate stability and structure, in addition to increasing total porosity and water-holding capacity. The application of different types of biochar significantly affects the soil aggregation indices^13^. Applying biochar to the soils significantly increased organic matter, cation exchange capacity, and the availability of nutrients necessary for plant growth as well as improved microbial activity and enzyme activity^14,15^. The properties of biochar control its effect on the salinity and other chemical properties of salt-affected soils. Notably, biochar feedstock is a main factor influencing electrical conductivity in soil after biochar applications^7^. Applying biochar to salt-affected soil caused significant decreases in electrical conductivity compared to unamended soil^16^. On the other hand, some studies have also found increased soil salinity and sodicity under applying biochar at high doses^17,18^. Biochar can decrease soil salinity via several mechanisms such as adsorbing salt ions and improving porosity and hydraulic conductivity, thus promoting salt leaching^19^. Biochar application to salt-affected soils improves plant growth through several mechanisms such as reducing oxidative stress, alleviating osmotic stress by increasing water holding capacity and availability, reducing plant hormone production, enhancing stomatal density and conductivity, and improving seed germination^20^. Applying biochar to saline soil improved soil quality indicators, alleviated soil salt stress, and primarily increased crops’ biomass^21,22^. Biochar improves hormonal and root anatomical characteristics under salt stress consequently increasing mung bean growth with greenhouse experiment conditions^23^.

Arugula (*Eruca sativa Mill.*) is an annual herbaceous plant from leafy vegetables belonging to the *Brassicaceae* family, which is cultivated in the Mediterranean basin. Arugula is used in traditional medicine because of its healing properties as an astringent, aphrodisiac, diuretic, digestive, emollient, tonic, depurative, laxative, rubefacient, and stimulant as well as used in the human diet^24^. Arugula leaves are used as a green salad and its seeds are a rich source of protein and oil^25^. Moreover, the arugula plant is also important because its application to food conservation and innovative sustainable nano-drugs, give the remarkable biological significance of its phytoactive compounds^26^. Arugula plant is cultivated in arid, semiarid regions and severely salt-affected soils. The salt tolerance of the arugula plant is attributed to its ability to exclude sodium, high selectivity for potassium: sodium ratio, and high uptake of calcium^25^. Arugula is a forgotten plant having a high tolerance to some abiotic stresses such as osmotic and salinity stresses, so arugula has a great opportunity to be used in agronomic and breeding programs in areas affected by drought stress^27^. Arugula has a rapid growth cycle; allowing farmers to achieve a high financial return even in marginal soil conditions^28^. Arugula is relatively salt-tolerant compared to many other crops^27^. Increasing salinity levels in the soil decreased the fresh biomass of the arugula plant^29^. Using poor-quality irrigation water and marginal soils in the presence of biochar to cultivate fresh vegetables such as arugula, having high nutritional value for human health, represents an interesting objective in modern agriculture. Several previous studies have focused on the effect of biochar on soils where no crops have been cultivated. In this study, we hypothesized that biochar prepared from different feedstock would have different positive effects on nutrient availability, soil characteristics, and growth parameters of arugula plant in saline soil under saline water irrigation. The feedstocks selected in this study for biochar production are commonly available locally, and the mismanagement of these wastes represents an environmental and health problem, contributing to environmental pollution and the spread of diseases. The objectives of this pot experiment were to investigate the effects of four types of biochar applied to saline soil at different doses under irrigation with saline water on: (1) soil chemical properties, (2) nutrient availability, and (3) growth parameters of arugula plant. We selected the pot experiment to achieve these objectives because it is a quick, simple, and controlled method for studying the effect of amendments on soil properties and plant growth.

## Materials and methods

### Biochar production

Four plant residues were selected: banana leaves, rice straw, sorghum stalks, and wood chips. Banana leaves were collected from a farmer in Assiut Governorate, Egypt. Rice straw and sorghum stalks were taken from the Faculty of Agriculture farm, Assiut University, Egypt. Wood chips were collected from a carpentry workshop at Assiut City, Egypt. Each plant residue was placed in an iron plate (33.5 * 22.6 * 22.6 cm) and burned at 300 °C for five hours in the oven under oxygen-limited conditions. The biochar production process was carried out as described by Amin^30^. After completing the burning process, the produced biochar was ground using a steel grinder having a sieve with a hole diameter of 1 mm. The pH of biochar was measured in a 1:10 ratio of biochar to distilled water suspensions using a glass electrode. However, the electrical conductivity (EC) of different biochar types was measured in a 1:10 ratio of biochar to distilled water extracts using an EC meter^31^. Cation exchange capacity (CEC) was determined in the biochar according to Domingues et al.^32^. The content of carbon, hydrogen, nitrogen, and sulfur in biochar was determined using a CHNS elemental analyzer (Elementar Vario EL, Germany). Important properties of biochar are shown in Table 1.

**Table 1:** Some important properties of wood chips Biochar, banana leaves Biochar, sorghum stalks Biochar, and rice straw Biochar.

Property	Unit	Wood chips biochar	Banana leaves biochar	Sorghum stalks biochar	Rice straw biochar
pH (1:10)		5.63 ± 0.11	6.69 ± 0.01	7.08 ± 0.02	6.72 ± 0.03
EC (1:10)	dS m^− 1^	0.68 ± 0.04	9.89 ± 0.01	12.26 ± 0.06	10.29 ± 0.26
CEC	cmol + kg^− 1^	13.08 ± 0.13	30.73 ± 0.83	30.33 ± 1.00	25.59 ± 1.10
C	%	67.79	50.5	52.73	44.08
N	%	0.47	1.33	1.154	1.273
S	%	0.378	0.36	0.387	0.421
H	%	1.828	0.468	1.27	1.066
C/N ratio	-	144.23	37.97	45.69	34.63
C/S ratio	-	179.34	140.28	136.25	104.70
H/C ratio	-	0.03	0.01	0.02	0.02
N/C ratio	-	0.007	0.026	0.022	0.029

EC: Electrical conductivity; CEC: cation exchange capacity; C: carbon; N: nitrogen; S: sulfur; H: hydrogen.

### Open pot experiment

Soil samples were collected from the Extension Farm, Faculty of Agriculture, Assiut University, Assiut, Egypt. The collected soil samples were air-dried and passed through a 2 mm sieve. The soil used in this pot experiment was classified according to US soil taxonomy as Entisols; Typic Torripsamments. The important physico-chemical properties of the soil under study are shown in Table 2. In the current experiment, four types of biochar: banana leaves biochar (BLB), rice straw biochar (RSB), sorghum stalks biochar (SSB), and wood chips biochar (WCB), were applied to the soil in the pots at levels of 1%, 3%, and 5% (w/w). The open pot experiment included 13 treatments: (1) Control (unamended soil), (2) soil amended with 1% WCB (1%WCB ), (3) soil amended with 3% WCB (3%WCB ), (4) soil amended with 5% WCB (5%WCB ), (5) soil amended with 1% BLB (1%BLB), (6) soil amended with 3% BLB (3%BLB), (7) soil amended with 5% BLB (5%BLB), (8) soil amended with 1% SSB (1%SSB), (9) soil amended with 3% SSB (3%SSB), (10) soil amended with 5% SSB (5%SSB), (11) soil amended with 1% RSB (1%RSB), (12) soil amended with 3% RSB (3%RSB), and (13) soil amended with 5% RSB (5%RSB). Each round plastic pot (14 cm height x 11.5 cm base diameter x 15 cm top diameter) was filled with 2 kg of the soil. Round plastic pots have holes at the bottom to drain excess water. Ten arugula seeds were planted in each pot on November 6, 2022. This experiment was irrigated with saline water with an electrical conductivity of 6.2 dS m^− 1^, conducted in a completely randomized design with three replications, and placed in an open environment. Then the plants were thinned to four plants in the pot after 17 days of planting. Saline water used in this experiment was prepared from sodium chloride and calcium chloride in a ratio of 1:2, respectively. Nitrogen fertilizer was applied in solution form to all pots at level 335 mg N per pot. The nitrogen was added in two doses. The chlorophyll value was measured by SPAD-502 in the arugula plant after 68 days from planting. After 73 days from planting, the arugula was harvested on January 17, 2023. Then, fresh biomass of shoots was recorded per pot. The shoot of arugula was washed with distilled water and oven-dried at 70 °C until the weight was stable. The weight of the dry biomass of shoots was recorded. Soil samples were collected from each pot after harvesting arugula, air-dried, crushed, and kept for chemical analysis. This experiment was carried out at the Soils and Water Department, Faculty of Agriculture, Assiut University, Assiut, Egypt.

**Table 2:** Some physical and chemical properties of the saline sandy soil. Each value ± standard deviation.

Property	Unit	Value ± SD
Sand	%	93.6 ± 0.00
Silt	%	3.8 ± 0.28
Clay	%	2.6 ± 0.28
Texture		Sand
CaCO_3_	%	24.25 ± 3.54
C	%	1.268
N	%	0.086
S	%	0.272
H	%	0.38
EC_(1:1)_	dS m^‒1^	4.12 ± 0.01
Soluble calcium	mmol kg^− 1^	14.33 ± 0.24
Soluble magnesium	mmol kg^− 1^	1.42 ± 0.35
Soluble sodium	mmol kg^− 1^	2.42 ± 0.08
Soluble potassium	mmol kg^− 1^	1.77 ± 0.04
Soluble chloride	mmol kg^− 1^	3.11 ± 0.00
Soluble bicarbonate	mmol kg^− 1^	0.94 ± 0.17
Soluble sulfate	mmol kg^− 1^	13.32 ± 0.50
Available phosphorus	mg kg^− 1^	1.93 ± 0.49
Available potassium	mmol kg^− 1^	5.50 ± 0.44

C: carbon; N: nitrogen; S: sulfur; H: hydrogen; EC: Electrical conductivity.

### Soil analyses

The particle size distribution of the soil under study was estimated by the pipette method^33^. Calcium carbonate in the soil was estimated by the calcimeter method^34^. Extracts of soil samples were prepared at a ratio of 1:1 soil (g) to distilled water (ml). Electrical conductivity (EC) in soil extract was measured via an electrical conductivity meter^35^. Soluble Ca and Mg in the soil extracts were estimated by Na_2_EDTA solution (disodium ethylene diamine tetra-acetic acid); soluble K and Na were measured by flame photometer. Soluble bicarbonate (HCO_3_) + carbonate (CO_3_) was determined by HCl acid, soluble sulfate was estimated by the turbidimetry method using barium chloride^36^, and soluble chloride was estimated by silver nitrate solution^35^. Soil organic matter was estimated using the dichromate oxidation method^37^. Cation exchange capacity (CEC) was determined in the soil samples after harvesting the arugula plant using 1 M sodium acetate at pH 8.2^36^. The total available nitrogen in the soil was extracted by 1 M KCl^38^. The total available nitrogen including ammonium nitrogen and nitrate nitrogen in the soil extracts was determined by the Kjeldahl method in two steps: estimation of ammonium alone in the soil extracts and nitrate in the soil extracts by adding Devarda’s alloy to convert the entire nitrate into ammonium^39^. Soil-available phosphorus was extracted by 0.5 M sodium bicarbonate with pH 8.5^40^ and phosphorus in soil extracts was analyzed by colorimetric method^35^. Available potassium (K) in soil samples was extracted with 1 M ammonium acetate, pH 7, and then determined by a flame photometer^36^.

### Plant analysis

Arugula shoot samples were digested by a mixture of H_2_SO_4_-H_2_O_2_ to determine the total content of nitrogen, phosphorus, potassium, and sodium. We took o.4 g of the plant sample, placed it in the digestion unit tube, added the digestion mixture, and left the sample overnight. The digestion process of plant samples was done in a closed vessel in the digestion unit. At the beginning of the digestion process, the temperature started at 120 ºC, then the temperature was gradually raised to reach 360 ºC until the digestion process was completed and the solution became clear white^41^. The total nitrogen in all digests was determined by the micro-Kjeldahl method and phosphorus was measured colorimetrically by the chlorostannous phosphomolybdic acid method in the sulphuric acid system^35^, also, potassium and sodium were analyzed by flame photometry method. The nutrient uptake (mg pot^− 1^) in arugula shoot was calculated by the following equation^10^:

$$\:\text{N}\text{u}\text{t}\text{r}\text{i}\text{e}\text{n}\text{t}\:uptake\:\left(mg\:{pot}^{-1}\right)=\frac{\text{N}\text{u}\text{t}\text{r}\text{i}\text{e}\text{n}\text{t}\:\text{c}\text{o}\text{n}\text{c}\text{e}\text{n}\text{t}\text{r}\text{a}\text{t}\text{i}\text{o}\text{n}\:\left(\text{m}\text{g}\:{kg}^{-1}\right)\:\text{i}\text{n}\:\text{d}\text{r}\text{y}\:\text{b}\text{i}\text{o}\text{m}\text{a}\text{s}\text{s}\:\text{X}\:\text{w}\text{e}\text{i}\text{g}\text{h}\text{t}\:\text{o}\text{f}\:\text{d}\text{r}\text{y}\:\text{b}\text{i}\text{o}\text{m}\text{a}\text{s}\text{s}\:\left(\text{g}\:{pot}^{-1}\right)}{1000}$$

### Statistical analysis

The data in this study were statistically analyzed by a one-way analysis of variance (ANOVA) using the MSTATC program. Significant differences among treatments were performed using Tukey’s honestly significant difference test (Tukey’s HSD) at a probability (p) of 0.01. The Pearson correlation analyses were carried out using the SRPLOT program.

## Results

### Electrical conductivity

**Fig. 1:**
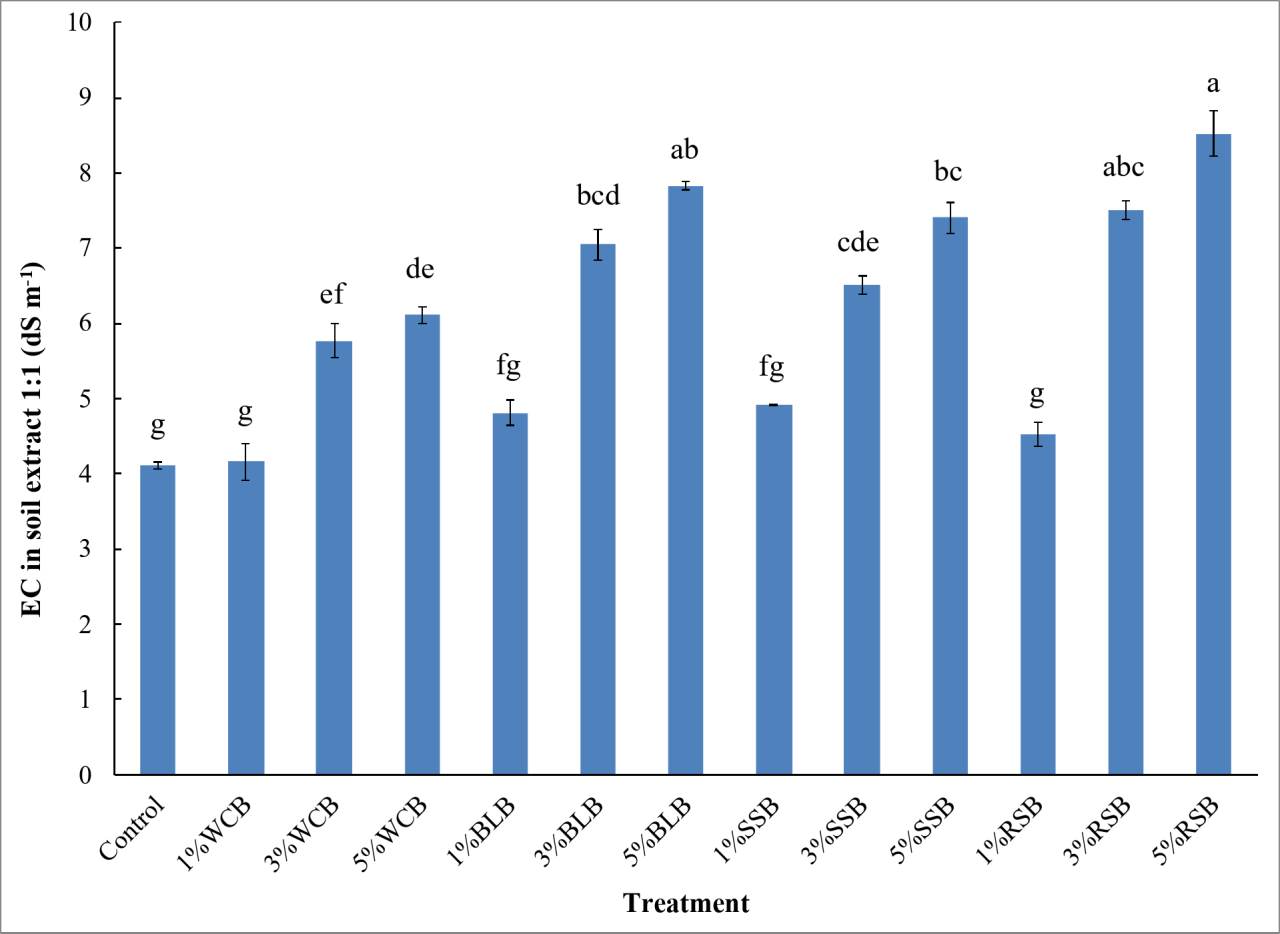
Electrical conductivity (EC) in extract 1:1 of saline sandy soil after harvesting arugula plant as affected by type and doses of biochar. Control: unamended soil; WCB, wood chips biochar; BLB: banana leaves biochar, SSB: sorghum stalks biochar, RSB: rice straw biochar. Biochar was applied at three doses 1%, 3%, and 5% (w/w). Different lowercase letters on each bar indicate the significant differences among treatments according to Tukey’s Honestly Significant Difference test at *p* ≤ 0.01. Vertical bars indicate the standard error of the mean (*n* = 3 replicates).

Under irrigation with saline water, the applications of 3%WCB, 5%WCB, 3%BLB, 5%BLB, 3%SSB, 5%SSB, 3%RSB, and 5%RSB in saline soil led to a significant increase (*p* ≤ 0.01) in the electrical conductivity (EC) values compared to the control treatment after harvesting arugula plant (Fig. 1). Compared with the control treatment, the EC increased by 1%, 40%, 49%, 17%, 71%, 91%, 20%, 59%, 80%, 10%, 83%, and 107% for 1%WCB, 3%WCB, 5%WCB, 1%BLB, 3%BLB, 5%BLB, 1%SSB, 3%SSB, 5%SSB, 1%RSB, 3%RSB, and 5%RSB treatments, respectively. The lowest value of EC was observed at the control treatment. However, the highest value was noticed in the 5%RSB treatment. The values of EC in saline soil increased with increasing biochar doses (Fig. 1).

### Soluble cations and anions

After the arugula plant was harvested, the addition of 3%WCB, 5%WCB, 1%BLB, 3%BLB, 1%SSB, 3%SSB, 5%SSB, 3%RSB, and 5%RSB treatments significantly increased soluble calcium in the soil under study compared with the control treatment (Table 3). Meanwhile, adding 1%WCB, 5%BLB, and 1%RSB showed non-significant increases in soluble calcium. The lowest concentration of soluble calcium was observed at the control treatment, while the highest concentration was observed when applying 3%RSB treatments. Compared with the control treatment, adding 3%BLB, 5%BLB, 3%SSB, and 5%SSB treatments to saline soil significantly increased soluble magnesium, while applying 1%WCB, 3%WCB, 5%WCB, 1%RSB, 3%RSB, and 5%RSB treatments showed non-significant increases in soluble magnesium. Applying 1%BLB and 1%SSB treatments to saline soil showed non-significant decreases in soluble magnesium (Table 3). The lowest concentration of soluble magnesium was observed at 1%BLB treatment, while the highest concentration was observed in the 5%BLB application. The application of 3%WCB, 5%WCB, 1%BLB, 3%BLB, 5%BLB, 1%SSB, 3%SSB, 5%SSB, 3%RSB, and 5%RSB treatments to sandy soil increased significantly soluble sodium compared to the control treatment after harvesting arugula plant (Table 3). However, adding 1%WCB and 1%RSB resulted in a non-significant increase in the soluble sodium. Soluble sodium increased relative to the control treatment by 19%, 254%, 230%, 64%, 250%, 253%, 118%, 271%, 285%, 36%, 327%, and 364% for 1%WCB, 3%WCB, 5%WCB, 1%BLB, 3%BLB, 5%BLB, 1%SSB, 3%SSB, 5%SSB, 1%RSB, 3%RSB, and 5%RSB, respectively. The lowest content of soluble sodium was observed in the control treatment. However, the highest content of soluble sodium was observed when adding 5%RSB. Applying 3%BLB, 5%BLB, 1%SSB, 3%SSB, 5%SSB, 1%RSB, 3%RSB, and 5%RSB to saline soil increased significantly soluble potassium in comparison with the control treatment (Table 3). Meanwhile, adding 1%WCB, 3%WCB, 5%WCB, and 1%BLB showed non-significant increases in soluble potassium. Soluble potassium increased relative to the control treatment by 1.1-fold, 1.3, 1.4, 2.3, 7.8, 11.7, 2.6, 7.2, 11.3, 2.6, 9.8, and 19.4-fold for 1%WCB, 3%WCB, 5%WCB, 1%BLB, 3%BLB, 5%BLB, 1%SSB, 3%SSB, 5%SSB, 1%RSB, 3%RSB, and 5%RSB, respectively. The concentrations of soluble potassium in saline soil increased with increasing biochar doses. The lowest value of soluble potassium was present at the control treatment, while the highest value was observed at the 5%RSB treatment.

**Table 3:** Effect of type and doses of Biochar on soluble cations and anions in soil extract 1:1 after harvesting arugula plant grown in saline sandy soil (data were mean ± standard deviation, (*n* = 3)).

Treatment	Soluble cations and anions in soil extract 1:1 (mmol kg^− 1^)						
	Calcium	Magnesium	Sodium	Potassium	bicarbonate	Chloride	Sulfate
Control	14.13 ± 0.61d	2.95 ± 0.37efg	4.36 ± 0.25f	1.17 ± 0.03f	1.55 ± 0.04f	6.29 ± 1.09 g	15.80 ± 0.37ef
1%WCB	15.72 ± 0.61bcd	3.56 ± 0.37defg	5.20 ± 1.66ef	1.27 ± 0.22ef	1.57 ± 0.10f	7.23 ± 0.31 fg	17.00 ± 1.17cdef
3%WCB	16.96 ± 0.92ab	3.50 ± 0.81defg	15.42 ± 0.84c	1.48 ± 0.02ef	2.37 ± 0.05def	20.44 ± 1.26de	15.35 ± 0.61f
5%WCB	18.20 ± 0.31a	4.41 ± 1.03cdef	14.40 ± 0.52c	1.63 ± 0.06ef	2.67 ± 0.54cde	24.74 ± 0.48 cd	15.97 ± 0.83def
1%BLB	16.61 ± 0.31abc	1.37 ± 0.02 g	7.17 ± 0.53de	2.73 ± 0.33ef	2.10 ± 0.18def	13.52 ± 2.18ef	16.97 ± 1.39cdef
3%BLB	16.61 ± 0.31abc	6.82 ± 1.50bc	15.28 ± 1.26c	9.08 ± 0.33d	2.99 ± 0.22bcd	28.20 ± 3.62c	19.67 ± 2.06 cd
5%BLB	15.55 ± 0.31bcd	9.63 ± 1.24a	15.38 ± 0.30c	13.69 ± 0.33b	4.47 ± 0.05a	30.71 ± 0.96c	27.11 ± 1.13a
1%SSB	16.61 ± 0.61abc	2.20 ± 0.55 fg	9.51 ± 0.01d	3.10 ± 0.19e	1.95 ± 0.09ef	14.15 ± 0.94ef	16.42 ± 1.41def
3%SSB	16.61 ± 0.81abc	5.56 ± 0.21bcd	16.19 ± 1.33bc	8.44 ± 0.02d	3.43 ± 0.65bc	28.82 ± 3.58c	20.72 ± 0.48bc
5%SSB	16.78 ± 0.31abc	7.97 ± 0.28ab	16.79 ± 0.34bc	13.25 ± 1.63bc	3.88 ± 0.52ab	31.76 ± 1.91bc	23.91 ± 1.15ab
1%RSB	14.84 ± 0.53 cd	3.25 ± 0.26defg	5.92 ± 0.16ef	3.08 ± 0.20e	1.78 ± 0.09ef	11.11 ± 2.90 fg	16.38 ± 0.43def
3%RSB	18.37 ± 0.81a	4.24 ± 0.44def	18.60 ± 0.66ab	11.50 ± 0.16c	2.69 ± 0.10cde	38.36 ± 2.20ab	19.35 ± 1.16cde
5%RSB	18.20 ± 0.61a	5.32 ± 0.66cde	20.23 ± 0.25a	22.70 ± 0.79a	3.46 ± 0.09abc	39.47 ± 0.16a	20.78 ± 0.71bc

Different lowercase letters within the same column indicate the significant differences among treatments according to Tukey’s Honestly Significant Difference test at *p* ≤ 0.01. Control: unamended soil; WCB, wood chips biochar; BLB: banana leaves biochar, SSB: sorghum stalks biochar, RSB: rice straw biochar. Biochar was applied at three doses 1%, 3%, and 5% (w/w).

Adding 5%WCB, 3%BLB, 5%BLB, 3%SSB, 5%SSB, 3%RSB, and 5%RSB to saline soil showed an increase significant in soluble bicarbonate compared to the control treatment (Table 3). Meanwhile, applying 1%WCB, 3%WCB, 1%BLB, 1%SSB, and 1%RSB showed non-significant increases in soluble bicarbonate. The lowest concentration of soluble bicarbonate was observed in the control treatment. However, the highest concentration of soluble bicarbonate was observed at 5%BLB treatment. The addition of 3%WCB, 5%WCB, 1%BLB, 3%BLB, 5%BLB, 1%SSB, 3%SSB, 5%SSB, 3%RSB, and 5%RSB treatments significantly increased soluble chloride in the soil under study compared with the control treatment (Table 3). Meanwhile, adding 1%WCB and 1%RSB showed non-significant increases in soluble chloride. The lowest concentration of soluble chloride was observed in the control treatment. However, the highest concentration of soluble chloride was observed when applying 5%RSB treatment. Applying 3%BLB, 5%BLB, 3%SSB, 5%SSB, and 5%RSB treatments to saline soil significantly increased soluble sulfate compared to the control treatment after harvesting the arugula plant (Table 3). However, applying 1%WCB, 5%WCB, 1%BLB, 1%SSB, 1%RSB, and 3%RSB to saline soil led to non-significant increases in soluble sulfate. Addition of 3%WCB to saline soil led to a non-significant decrease in soluble sulfate. The lowest concentration of soluble sulfate was observed when adding 3%WCB treatment, whilst the highest concentration of soluble sulfate was observed when applying 5%BLB treatment (Table 3).

### Soil organic matter and cation exchange capacity

Compared to the control treatment, all biochar treatments applied to saline soil resulted in significant increases in the soil organic matter (SOM) after harvesting arugula, except for the 1%WCB treatment which showed a non-significant increase (Table 4). The contents of SOM in saline soil increased with increasing biochar doses. Adding biochar to the saline soil contributed to increasing the organic matter by 7.0, 16.0, 30.0, 14.0, 33.9, 51.8, 13.6, 35.7, 50.2, 12.3, 32.7, and 66.2-fold higher than the control treatment for 1%WCB, 3%WCB, 5%WCB, 1%BLB, 3%BLB, 5%BLB, 1%SSB, 3%SSB, 5%SSB, 1%RSB, 3%RSB, and 5%RSB treatments, respectively. The effectiveness of the treatments in this study on the SOM increase was in the order of 5%RSB > 5%BLB > 5%SSB > 3%SSB > 3%BLB > 3%RSB > 5%WCB > 3%WCB > 1%BLB > 1%SSB > 1%RSB > 1%WCB > control. The control treatment had the lowest SOM content. However, the 5%RSB treatments had the highest content (Table 4). Significantly increased the cation exchange capacity (CEC) resulting in the applications of 3%WCB, 5%WCB, 3%BLB, 5%BLB, 3%SSB, 5%SSB, 3%RSB, and 5%RSB treatments to saline soil compared to the control treatment (Table 4). Adding all different types of biochar at levels of 3 and 5% led to a significant increase in the exchange capacity compared to the control treatment. However, applying 1%WCB, 1%BLB, 1%SSB, and 1%RSB treatments had a non-significant increase in CEC. The values of CEC in saline soil increased with increasing biochar doses. Relative to the control treatment, the CEC values increased by 7%, 26%, 22%, 3%, 30%, 58%, 6%, 31%, 54%, 7%, 28%, and 48% for 1%WCB, 3%WCB, 5%WCB, 1%BLB, 3%BLB, 5%BLB, 1%SSB, 3%SSB, 5%SSB, 1%RSB, 3%RSB, and 5%RSB, respectively. The CEC values in saline soil increased with increasing biochar doses. The lowest value of CEC was observed at the control treatment. However, the highest values were shown in the 5%BLB and 5%SSB treatments (Table 4).

**Table 4:** Effect of type and doses of Biochar on organic matter, cation exchange capacity, available nitrate, available ammonium, and total available nitrogen in saline sandy soil after harvesting arugula plant (data were mean ± standard deviation, (*n* = 3)).

Treatment	O.M (%)	CEC (cmol + kg^− 1^)	Available NO_3_-*N* (mg kg^− 1^)	Available NH_4_-*N* (mg kg^− 1^)	Total available *N* (mg kg^− 1^)
Control	0.06 ± 0.02f	3.19 ± 0.22 g	52.22 ± 5.22f	53.96 ± 3.01ab	106.18 ± 3.01de
1%WCB	0.42 ± 0.06ef	3.41 ± 0.20defg	70.50 ± 2.61de	50.48 ± 3.01b	120.98 ± 3.99 cd
3%WCB	0.96 ± 0.08d	4.02 ± 0.17cde	87.90 ± 3.01abc	61.79 ± 3.99a	149.70 ± 1.51a
5%WCB	1.80 ± 0.05c	3.88 ± 0.08cdef	88.77 ± 4.52abc	53.96 ± 3.01ab	142.74 ± 3.99ab
1%BLB	0.84 ± 0.14d	3.29 ± 0.07 fg	96.61 ± 2.61ab	54.83 ± 4.52ab	151.44 ± 6.91a
3%BLB	2.03 ± 0.22c	4.15 ± 0.09bc	87.90 ± 3.01abc	54.83 ± 4.52ab	142.74 ± 3.99ab
5%BLB	3.11 ± 0.20b	5.05 ± 0.06a	87.03 ± 1.51bc	53.96 ± 3.01ab	140.99 ± 2.61ab
1%SSB	0.82 ± 0.05de	3.38 ± 0.04efg	60.05 ± 0.00ef	53.09 ± 1.51ab	113.14 ± 1.51de
3%SSB	2.14 ± 0.08c	4.17 ± 0.12bc	53.53 ± 6.53f	52.22 ± 0.00b	105.75 ± 6.53e
5%SSB	3.01 ± 0.18b	4.90 ± 0.54a	79.64 ± 6.53 cd	52.22 ± 0.00b	131.86 ± 6.53bc
1%RSB	0.74 ± 0.03de	3.42 ± 0.17defg	97.48 ± 1.51ab	52.22 ± 0.00b	149.70 ± 1.51a
3%RSB	1.96 ± 0.05c	4.08 ± 0.10bcd	65.28 ± 5.22ef	52.22 ± 0.00b	117.50 ± 5.22cde
5%RSB	3.97 ± 0.16a	4.72 ± 0.10ab	100.96 ± 1.51a	52.22 ± 0.00b	153.18 ± 1.51a

O.M: organic matter; CEC: cation exchange capacity; NO_3_-N: nitrate nitrogen; NH_4_-N: ammonium nitrogen. Different lowercase letters within the same column indicate the significant differences among treatments according to Tukey’s Honestly Significant Difference test at *p* ≤ 0.01. Control: unamended soil; WCB, wood chips biochar; BLB: banana leaves biochar, SSB: sorghum stalks biochar, RSB: rice straw biochar. Biochar was applied at three doses 1%, 3%, and 5% (w/w).

### Nutrient availability in saline soil

Applying 1%WCB, 3%WCB, 5%WCB, 1%BLB, 3%BLB, 5%BLB, 5%SSB, 1%RSB, and 5%RSB treatments increased significantly available nitrate nitrogen in the soil under study compared with the control treatment after harvesting arugula. Meanwhile, adding 1%SSB, 3%SSB, and 3%RSB showed non-significant increases in available nitrate nitrogen (Table 4). Relative to the control treatment, the available nitrate nitrogen in the soil increased by 35%, 68%, 70%, 85%, 68%, 67%, 15%, 3%, 53%, 87%, 25%, and 93% for 1%WCB, 3%WCB, 5%WCB, 1%BLB, 3%BLB, 5%BLB, 1%SSB, 3%SSB, 5%SSB, 1%RSB, 3%RSB, and 5%RSB, respectively. The effectiveness of the treatments for the increase in available nitrate nitrogen values was in the order: 5%RSB > 1%RSB > 1%BLB > 5%WCB > 3%WCB > 3%BLB > 5%BLB > 5%SSB > 1%WCB > 3%RSB > 1%SSB > 3%SSB > control. The lowest concentration of available nitrate nitrogen was observed at the control treatment. However, the highest concentration was shown in the 5%RSB treatment (Table 4). The results obtained from this experiment also resulted in a non-significant increase in the available ammonium nitrogen by applying 3%WCB, 1%BLB, and 3%BLB treatments to saline soil compared to the control treatment, but adding 1%WCB, 1%SSB, 3%SSB, 5%SSB, 1%RSB, 3%RSB, and 5%RSB treatments caused a non-significant decrease in available ammonium nitrogen content compared to the control treatment (Table 4). Adding 3%WCB, 5%WCB, 1%BLB, 3%BLB, 5%BLB, 5%SSB, 1%RSB, and 5%RSB treatments caused a significant increase in the total available nitrogen (nitrate nitrogen + ammonium nitrogen) in the soil under study compared with the control treatment (Table 4). However, adding 1%WCB, 1%SSB, and 3%RSB resulted in a non-significant increase in total available nitrogen. On the other hand, applying 3%SSB to saline soil led to a non-significant decrease in total available nitrogen compared to the control treatment. Relative to the control treatment, the total available nitrogen in the soil increased by 14%, 41%, 34%, 43%, 34%, 33%, 7%, 24%, 41%, 11%, and 44% for 1%WCB, 3%WCB, 5%WCB, 1%BLB, 3%BLB, 5%BLB, 1%SSB, 5%SSB, 1%RSB, 3%RSB, and 5%RSB, respectively. The treatments under study can be ranked in the total available nitrogen increment of this saline soil in the order of 5%RSB > 1%BLB > 3%WCB > 1%RSB > 5%WCB > 3%BLB > 5%BLB > 5%SSB > 1%WCB > 3%RSB > 1%SSB > control > 3%SSB (Table 4). The lowest concentration of total available nitrogen was observed at the 3%SSB treatment. However, the highest concentration was shown in the 5%RSB treatment (Table 4).

**Fig. 2:**
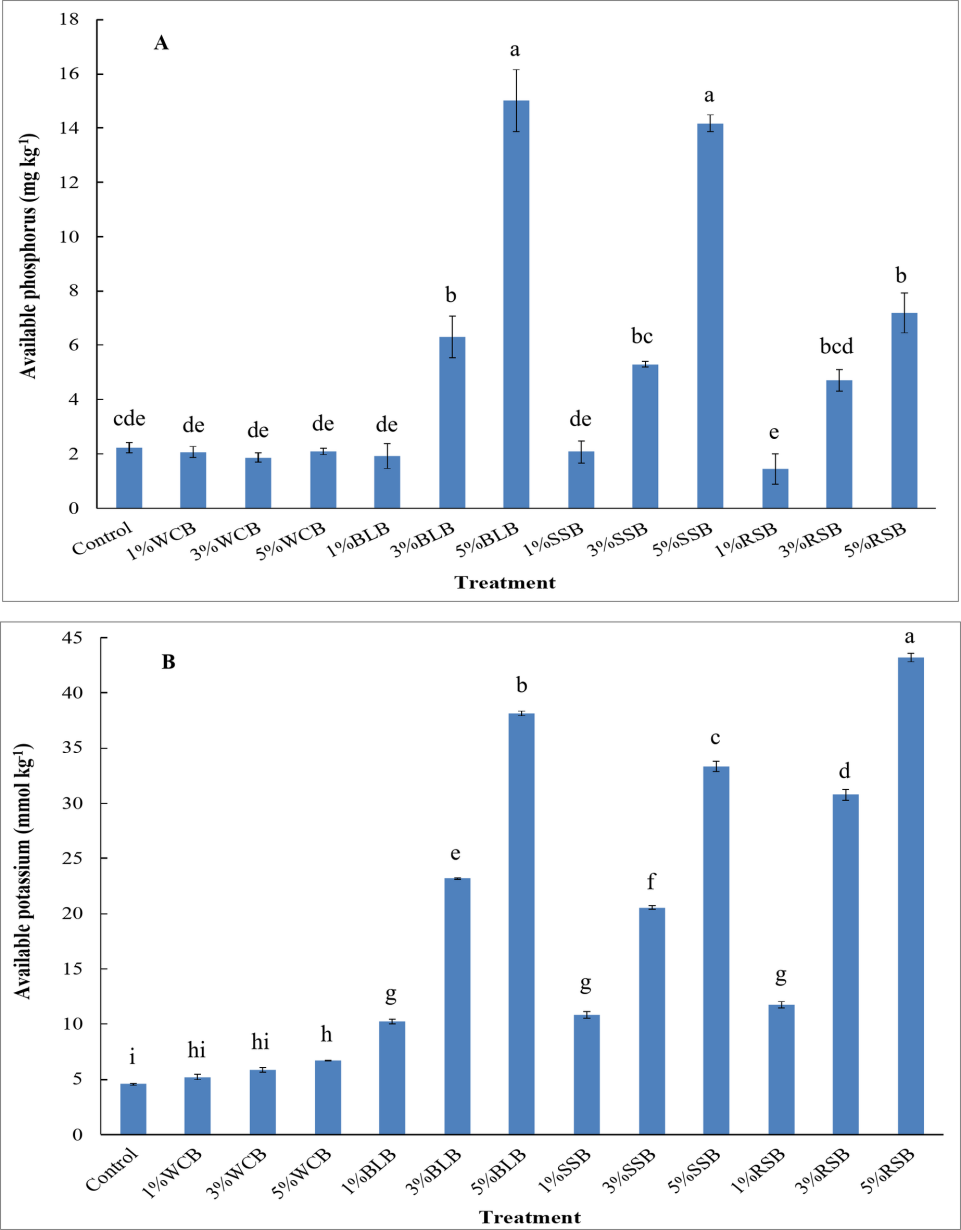
Available phosphorus and potassium in saline sandy soil after harvesting arugula plant as affected by type and doses of biochar. Control: unamended soil; WCB, wood chips biochar; BLB: banana leaves biochar, SSB: sorghum stalks biochar, RSB: rice straw biochar. Biochar was applied at three doses 1%, 3%, and 5% (w/w). Different lowercase letters on each bar indicate the significant differences among treatments according to Tukey’s Honestly Significant Difference test at *p* ≤ 0.01. Vertical bars indicate the standard error of the mean (*n* = 3 replicates).

After harvesting the arugula plant, the applications of 3%BLB, 5%BLB, 5%SSB, and 5%RSB treatments to saline soil significantly increased phosphorous availability compared to the control treatment (Fig. 2A). While applying 3%SSB and 3%RSB showed non-significant increases in phosphorous availability. On the other hand, adding 1%WCB, 3%WCB, 5%WCB, 1%BLB, 1%SSB, and 1%RSB, 3%RSB showed non-significant decrease in the phosphorous availability. Compared with the control treatment, the available phosphorus increased by 182%, 573%, 137%, 536%, 111%, and 222% for 3%BLB, 5%BLB, 3%SSB, 5%SSB, 3%RSB, and 5%RSB treatments, respectively. The effectiveness of the treatments in this study on the available phosphorus improvement was in the order of 5%BLB > 5%SSB > 5%RSB > 3%BLB > 3%SSB > 3%RSB > control > 1%SSB = 5%WCB > 1%WCB > 1%BLB > 3%WCB > 1%RSB (Fig. 2A). The lowest concentration of available phosphorus was observed at the 1%RSB treatment. Nevertheless, the highest concentration of available phosphorus was observed in saline soil with adding 5%BLB treatment.

Potassium availability in saline soil increased significantly after the harvesting of arugula plant under applying 5%WCB, 1%BLB, 3%BLB, 5%BLB, 1%SSB, 3%SSB, 5%SSB, 1%RSB, 3%RSB, and 5%RSB compared to the control treatment (Fig. 2B). However, applying 1%WCB and 3%WCB resulted in a non-significant increase in potassium availability. The applied biochar treatments increased available K over the control by more pronounced percentages reaching 14%, 29%, 48%, 125%, 410%, 738%, 137%, 352%, 632%, 158%, 576%, and 849% for 1%WCB, 3%WCB, 5%WCB, 1%BLB, 3%BLB, 5%BLB, 1%SSB, 3%SSB, 5%SSB, 1%RSB, 3%RSB, and 5%RSB treatments, respectively. The effectiveness of the treatments in this study on the available potassium increase was in the order of 5%RSB > 5%BLB > 5%SSB > 3%RSB > 3%BLB > 3%SSB > 1%RSB > 1%SSB > 1%BLB > 5%WCB > 3%WCB > 1%WCB > control (Fig. 2B). The lowest concentration of available potassium was observed in the control treatment, whilst the highest value of available potassium in saline soil was noticed in the 5%RSB treatment.

### Parameters growth of arugula plant

**Fig. 3:**
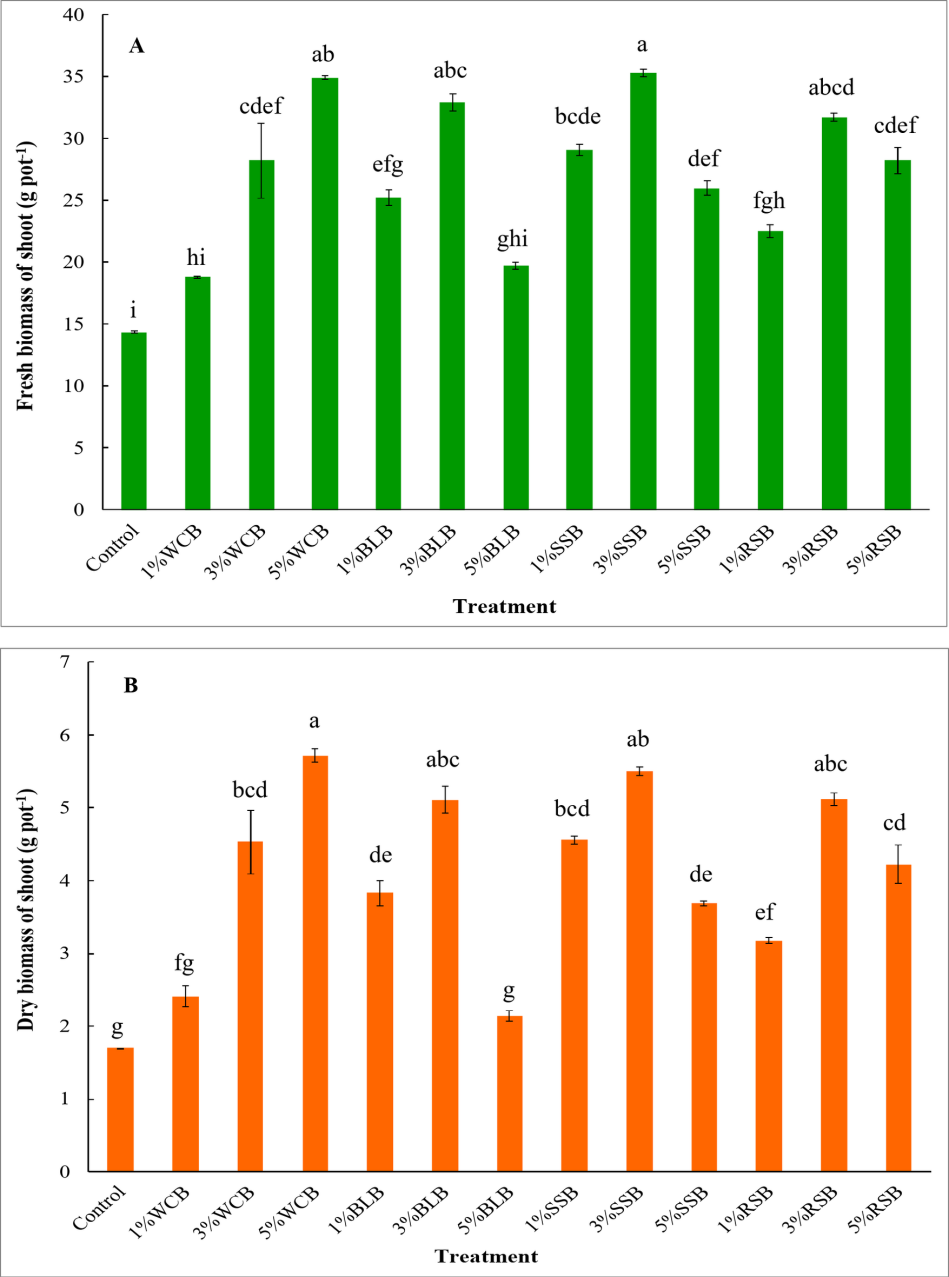
Fresh and dry biomass of arugula plant grown in saline sandy soil as affected by type and doses of biochar. Control: unamended soil; WCB, wood chips biochar; BLB: banana leaves biochar, SSB: sorghum stalks biochar, RSB: rice straw biochar. Biochar was applied at three doses 1%, 3%, and 5% (w/w). Different lowercase letters on each bar indicate the significant differences among treatments according to Tukey’s Honestly Significant Difference test at *p* ≤ 0.01. Vertical bars indicate the standard error of the mean (*n* = 3 replicates).

The results obtained from this experiment also resulted in a non-significant increase in the chlorophyll values under applying all treatments to the soil except 5%BLB treatment compared to the control treatment, but adding 5%BLB treatment showed a non-significant decrease in chlorophyll value compared to the control treatment (Table 5). A significant increase was observed in fresh and dry biomass of arugula plant grown in saline soil under applying 3%WCB, 5%WCB, 1%BLB, 3%BLB, 1%SSB, 3%SSB, 5%SSB, 1%RSB, 3%RSB, and 5%RSB treatments compared to the control treatment (Fig. 3A, B). However, adding 1%WCB and 5%BLB resulted in a non-significant increase in the fresh and dry biomass of the arugula plant. The relative increase in the fresh biomass of the arugula plant over the control treatment was 31%, 97%, 143%, 76%, 129%, 37%, 103%, 146%, 81%, 57%, 121%, and 97% for 1%WCB, 3%WCB, 5%WCB, 1%BLB, 3%BLB, 5%BLB, 1%SSB, 3%SSB, 5%SSB, 1%RSB, 3%RSB, and 5%RSB, respectively. The effectiveness of treatments in improving fresh biomass of arugula was in the order of 3%SSB > 5%WCB > 3%BLB > 3%RSB > 1%SSB > 5%RSB ≈ 3%WCB > 5%SSB > 1%BLB > 1%RSB > 5%BLB > 1%WCB > control (Fig. 3A). Relative to the control treatment, the dry biomass of the arugula plant increased by 42%, 166%, 236%, 125%, 201%, 26%, 168%, 224%, 117%, 87%, 201%, and 148% for 1%WCB, 3%WCB, 5%WCB, 1%BLB, 3%BLB, 5%BLB, 1%SSB, 3%SSB, 5%SSB, 1%RSB, 3%RSB, and 5%RSB, respectively. The treatments used in this soil showed improvements in the dry biomass of the arugula plant in the order of 5%WCB > 3%SSB > 3%RSB ≈ 3%BLB > 1%SSB > 3%WCB > 5%RSB > 1%BLB > 5%SSB > 1%RSB > 1%WCB > 5%BLB > control (Fig. 3B). The highest values of fresh and dry biomass of arugula plant were observed at the 5%WCB and 3%SSB applications, while the lowest values of fresh and dry biomass of arugula in this study were recorded for the control treatment.

**Table 5:** Effect of type and doses of Biochar on chlorophyll and nutrient content in arugula plant grown in saline sandy soil (data were mean ± standard deviation (*n* = 3)).

Treatment	Chlorophyll	Nutrient content in arugula plant (g kg^− 1^ plant)				K: Na ratio
		Nitrogen	Phosphorus	Potassium (K)	Sodium (Na)	
Control	48.36 ± 3.44ab	2.82 ± 0.00e	0.67 ± 0.02 g	15.05 ± 1.11e	5.97 ± 0.45b	2.53 ± 0.19 h
1%WCB	55.13 ± 3.00ab	3.15 ± 0.11de	0.94 ± 0.11efg	15.31 ± 2.46e	4.7 ± 0.53cde	3.26 ± 0.42gh
3%WCB	55.53 ± 2.45ab	5.86 ± 0.43a	0.92 ± 0.03efg	16.73 ± 0.09e	4.34 ± 0.11cde	3.86 ± 0.12defgh
5%WCB	55.30 ± 1.93ab	4.77 ± 0.00abc	1.22 ± 0.06d	17.04 ± 0.43e	3.93 ± 0.11de	4.34 ± 0.20cdefg
1%BLB	54.38 ± 3.89ab	5.06 ± 0.33abc	1.12 ± 0.02de	15.03 ± 0.02e	4.31 ± 0.23cde	3.50 ± 0.19efgh
3%BLB	55.66 ± 3.21ab	4.70 ± 0.13bc	1.01 ± 0.05def	23.83 ± 2.24 cd	5.00 ± 0.49bcd	4.78 ± 0.42cdef
5%BLB	44.64 ± 3.11b	4.70 ± 0.45bc	2.34 ± 0.05a	94.01 ± 0.40a	8.91 ± 0.24a	10.55 ± 0.30b
1%SSB	51.27 ± 4.78ab	5.43 ± 0.65ab	0.86 ± 0.07efg	18.7 ± 0.62de	3.57 ± 0.14e	5.25 ± 0.32 cd
3%SSB	51.48 ± 2.72ab	5.06 ± 0.25abc	1.92 ± 0.04b	24.37 ± 0.78 cd	4.55 ± 0.32cde	5.38 ± 0.46c
5%SSB	50.95 ± 2.33ab	4.63 ± 0.13bc	2.32 ± 0.07a	74.36 ± 0.64b	5.02 ± 0.23bcd	14.83 ± 0.80a
1%RSB	57.68 ± 0.40a	3.18 ± 0.45de	0.82 ± 0.03 fg	14.89 ± 0.05e	4.49 ± 0.12cde	3.32 ± 0.07fgh
3%RSB	50.21 ± 3.02ab	3.04 ± 0.22de	0.83 ± 0.14 fg	25.36 ± 1.13c	5.15 ± 0.54bc	4.96 ± 0.57cde
5%RSB	51.98 ± 5.33ab	4.12 ± 0.22 cd	1.56 ± 0.15c	75.07 ± 5.19b	5.18 ± 0.10bc	14.49 ± 0.72a

Different lowercase letters within the same column indicate the significant differences among treatments according to Tukey’s Honestly Significant Difference test at *p* ≤ 0.01. Control: unamended soil; WCB: wood chips biochar; BLB: banana leaves biochar; SSB: sorghum stalks biochar; RSB: rice straw biochar. Biochar was applied at three doses 1%, 3%, and 5% (w/w).

### The concentrations of nutrients in arugula plant

The applications of 3%WCB, 5%WCB, 1%BLB, 3%BLB, 5%BLB, 1%SSB, 3%SSB, 5%SSB, and 5%RSB to saline soil significantly increased nitrogen content in arugula plant compared with the control treatment. However, adding 1%WCB, 1%RSB, and 3%RSB treatments showed non-significant increases in nitrogen content in the arugula plant compared to the control treatment (Table 5). Adding 1%WCB, 3%WCB, 5%WCB, 1%BLB, 3%BLB, 5%BLB, 1%SSB, 3%SSB, 5%SSB, 1%RSB, 3%RSB, and 5%RSB treatments increased nitrogen concentration in arugula plant over the control by 12%, 108%, 69%, 80%, 67%, 67%, 92%, 80%, 64%, 13%, 8%, and 46%, respectively (Table 5). In this study, the effectiveness of the treatments in improving nitrogen content in arugula plant was in the order of 3%WCB > 1%SSB > 1%BLB = 3%SSB > 5%WCB > 3%BLB = 5%BLB > 5%SSB > 5%RSB > 1%RSB > 1%WCB > 3%RSB > control (Table 5). The lowest nitrogen content in the arugula plant was observed in the control treatment, whilst the highest nitrogen content in the arugula plant was noticed in the 3%WCB treatment.

A significant increase in phosphorus content occurred in arugula plant under applying 5%WCB, 1%BLB, 3%BLB, 5%BLB, 3%SSB, 5%SSB, and 5%RSB compared to the control treatment, on the other hand, the application of 1%WCB, 3%WCB, 1%SSB, 1%RSB, and 3%RSB caused an insignificant increase in phosphorus content in arugula plant (Table 5). Relative to the control treatment, phosphorus content in the arugula plant increased by 40%, 38%, 83%, 68%, 51%, 249%, 29%, 186%, 246%, 23%, 23%, 133% for the application of 1%WCB, 3%WCB, 5%WCB, 1%BLB, 3%BLB, 5%BLB, 1%SSB, 3%SSB, 5%SSB, 1%RSB, 3%RSB, and 5%RSB treatments, respectively. Therefore, all treatments increased the phosphorus content in the arugula plant in the order of 5%BLB > 5%SSB > 3%SSB > 5%RSB > 5%WCB > 1%BLB > 3%BLB > 1%WCB > 3%WCB > 1%SSB > 3%RSB > 1%RSB > control (Table 5). The lowest phosphorus content in the arugula plant was observed in the control treatment, whilst the highest phosphorus content in the arugula plant was noticed in the 5%BLB treatment.

The potassium content in the arugula plant grown in saline soil increased significantly with adding 3%BLB, 5%BLB, 3%SSB, 5%SSB, 3%RSB, and 5%RSB treatments compared with the control treatment. However, applying 1%WCB, 3%WCB, 5%WCB, and 1%SSB led to a non-significant increase in potassium content in the arugula plant. On the other hand, adding 1%BLB and 1%RSB showed a non-significant decrease in the potassium content in the arugula plant compared to the control treatment (Table 5). Relative to the control treatment, the potassium concentration in the arugula plant increased by 2%, 11%, 13%, 58%, 525%, 24%, 62%, 394%, 69%, and 399% with the addition of 1%WCB, 3%WCB, 5%WCB, 3%BLB, 5%BLB, 1%SSB, 3%SSB, 5%SSB, 3%RSB, and 5%RSB treatments, respectively. The effectiveness of treatments in improving potassium content in arugula was in the order of 5%BLB > 5%RSB > 5%SSB > 3%RSB > 3%SSB > 3%BLB > 1%SSB > 5%WCB > 3%WCB > 1%WCB > control > 1%BLB > 1%RSB (Table 5). The lowest potassium content in the arugula plant was observed in the 1%RSB treatment, whilst the highest potassium content in the arugula plant was noticed in the 5%BLB treatment.

Compared with the control treatment, adding 1%WCB, 3%WCB, 5%WCB, 1%BLB, 1%SSB, 3%SSB, and 1%RSB to saline soil significantly decreased the sodium content in the arugula plant. However, applying 3%BLB, 5%SSB, 3%RSB, and 5%RSB treatments resulted in a non-significant decrease in sodium content in the arugula plant (Table 5). Results showed that sodium concentration in the arugula plant decreased relative to the control treatment by 21%, 27%, 34%, 28%, 16%, 40%, 24%, 16%, 25%, 14%, and 13% for 1%WCB, 3%WCB, 5%WCB, 1%BLB, 3%BLB, 1%SSB, 3%SSB, 5%SSB, 1%RSB, 3%RSB, and 5%RSB treatments, respectively. On the other hand, applying 5%BLB to saline soil led to a significant increase in the sodium content in the arugula plant. Compared to the control, the 5%BLB addition increased sodium concentration in the arugula plant by 49%. The effectiveness of the treatments on sodium content in the arugula plant decrease was in the order of control > 5%RSB > 3%RSB > 5%SSB > 3%BLB > 1%WCB > 3%SSB > 1%RSB > 3%WCB > 1%BLB > 5%WCB > 1%SSB (Table 5). The lowest sodium content in the arugula plant was observed in the 1%SSB treatment, whilst the highest sodium content in the arugula plant was noticed in the 5%BLB treatment.

### Nutrient uptake by arugula plant

**Fig. 4:**
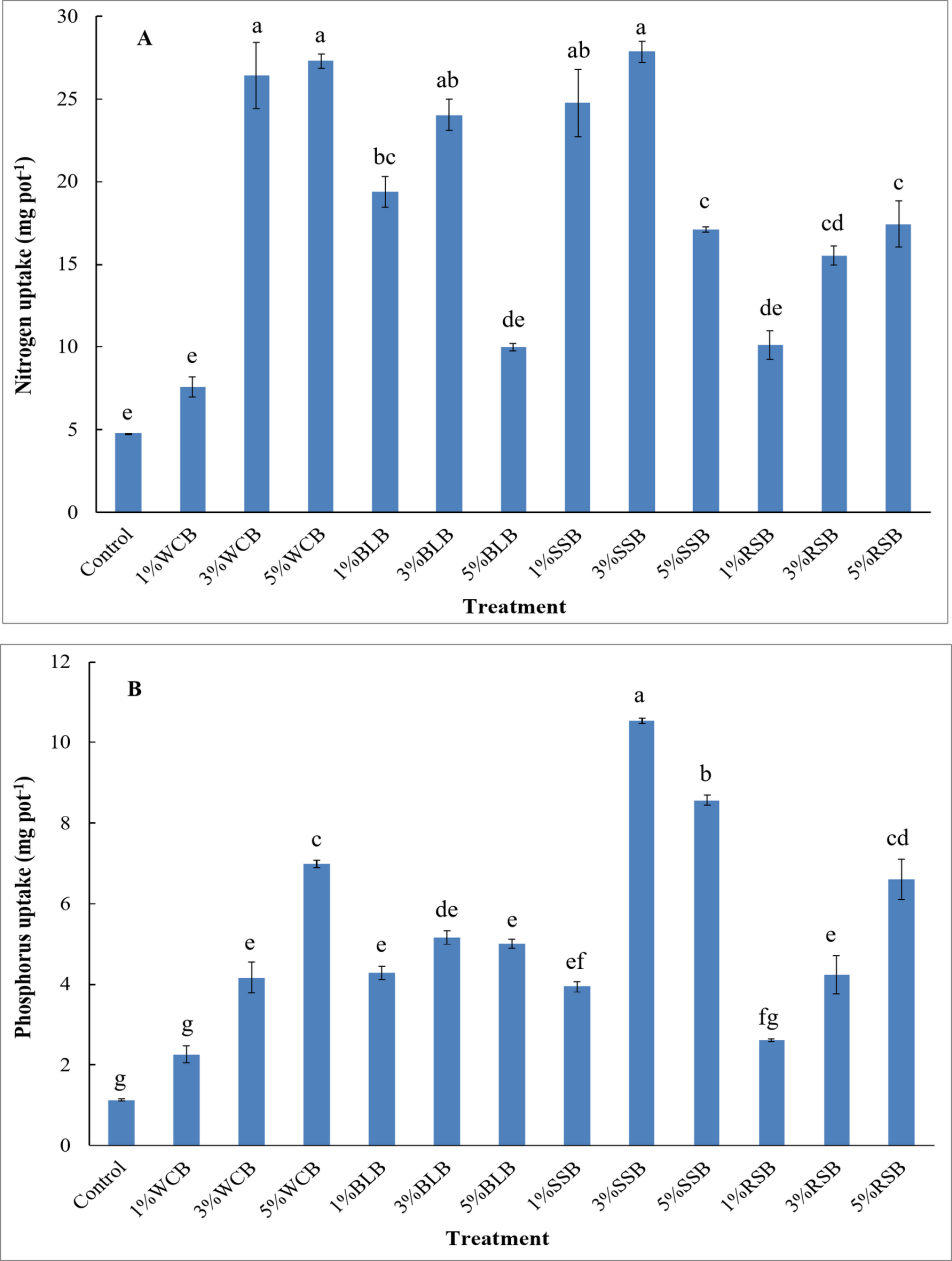
Nitrogen and phosphorus uptake by arugula plant grown in saline sandy soil as affected by type and doses of biochar; Control: unamended soil; WCB, wood chips biochar; BLB: banana leaves biochar, SSB: sorghum stalks biochar, RSB: rice straw biochar. Biochar was applied at three doses 1%, 3%, and 5% (w/w). Different lowercase letters on each bar indicate the significant differences among treatments according to Tukey’s Honestly Significant Difference test at *p* ≤ 0.01. Vertical bars indicate the standard error of the mean (*n* = 3 replicates).

A significant increase occurred in nitrogen uptake by arugula plant as a result of applying 3%WCB, 5%WCB, 1%BLB, 3%BLB, 1%SSB, 3%SSB, 5%SSB, 3%RSB, and 5%RSB treatments to saline soil compared to the control treatment. Meanwhile, applying 1%WCB, 5%BLB, and 1%RSB caused a non-significant increase in nitrogen uptake by the arugula plant (Fig. 4A). Results showed that the relative enhancements in of nitrogen uptake by arugula plant over the control were 59%, 453%, 471%, 305%, 403%, 110%, 418%, 483%, 257%, 112%, 225%, and 265% for 1%WCB, 3%WCB, 5%WCB, 1%BLB, 3%BLB, 5%BLB, 1%SSB, 3%SSB, 5%SSB, 1%RSB, 3%RSB, and 5%RSB treatments, respectively. The lowest nitrogen uptake by the arugula plant was observed in the control treatment, while the highest nitrogen uptake by the arugula plant was observed after adding 3%SSB treatment (Fig. 4A).

**Fig. 5:**
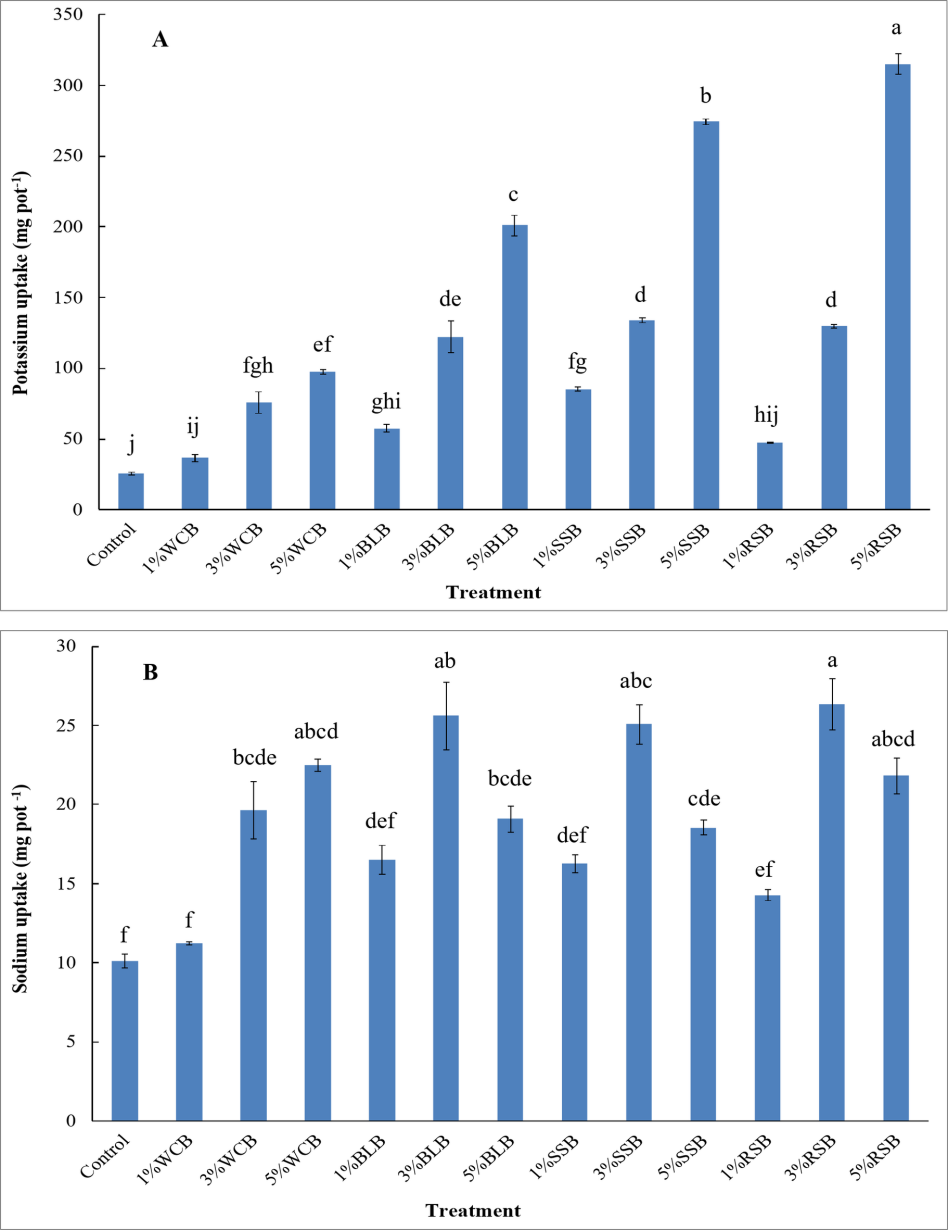
Potassium and sodium uptake by arugula plant grown in saline sandy soil as affected by type and doses of biochar. Control: unamended soil; WCB, wood chips biochar; BLB: banana leaves biochar, SSB: sorghum stalks biochar, RSB: rice straw biochar. Biochar was applied at three doses 1%, 3%, and 5% (w/w). Different lowercase letters on each bar indicate the significant differences among treatments according to Tukey’s Honestly Significant Difference test at *p* ≤ 0.01. Vertical bars indicate the standard error of the mean (*n* = 3 replicates).

Phosphorus and potassium uptake by arugula improved significantly with the applications of 3%WCB, 5%WCB, 1%BLB, 3%BLB, 5%BLB, 1%SSB, 3%SSB, 5%SSB, 3%RSB, and 5%RSB treatments to saline soil in comparison with the control treatment, while adding 1%WCB and 1%RSB treatments to this soil resulted in a non-significant increase in phosphorus and potassium uptake by the arugula plant compared to the control (Figs. 4B and 5A). Compared to the control, the biochar treatments of 1%WCB, 3%WCB, 5%WCB, 1%BLB, 3%BLB, 5%BLB, 1%SSB, 3%SSB, 5%SSB, 1%RSB, 3%RSB, and 5%RSB resulted in an increase in the phosphorus uptake by arugula plant by 100%, 269%, 518%, 280%, 357%, 343%, 248%, 833%, 657%, 132%, 275%, and 484%, respectively. The lowest phosphorus uptake by the arugula plant was observed in the control treatment, while the highest phosphorus uptake was noticed in the 3%SSB treatment (Fig. 4B). In addition, the relative increase in the potassium uptake by the arugula plant over the control was 44%, 197%, 282%, 126%, 379%, 688%, 234%, 425%, 975%, 86%, 408%, and 1135% for 1%WCB, 3%WCB, 5%WCB, 1%BLB, 3%BLB, 5%BLB, 1%SSB, 3%SSB, 5%SSB, 1%RSB, 3%RSB, and 5%RSB treatments, respectively. The lowest potassium uptake by the arugula plant was observed in the control treatment, while the highest potassium uptake was noticed when adding 5%RSB treatment (Fig. 5A).

Sodium uptake by the arugula plant increased significantly with the applications of 3%WCB, 5%WCB, 3%BLB, 5%BLB, 3%SSB, 5%SSB, 3%RSB, and 5%RSB treatments to saline soil compared to the control treatment, while the addition of 1%WCB,1%BLB, 1%SSB, and 1%RSB treatments led to no significant increase in the sodium uptake by the arugula plant (Fig. 5B). Relative to the control, the sodium uptake by the arugula plant increased by 11%, 94%, 122%, 63%, 153%, 89%, 61%, 148%, 83%, 41%, 160%, and 116% with the biochar treatments as 1%WCB, 3%WCB, 5%WCB, 1%BLB, 3%BLB, 5%BLB, 1%SSB, 3%SSB, 5%SSB, 1%RSB, 3%RSB, and 5%RSB treatments, respectively. The lowest sodium uptake by the arugula plant was observed in the control treatment, while the highest sodium uptake content was noticed after adding 3%RSB treatment (Fig. 5B).

Potassium: sodium ratio increased significantly in arugula plant resulted from the applications of 5%WCB, 3%BLB, 5%BLB, 1%SSB, 3%SSB, 5%SSB, 3%RSB, and 5%RSB treatments to saline soil compared to the control treatment. On the other hand, applying 1%WCB, 3%WCB, 1%BLB, and 1%RSB treatments showed a non-significant increase in potassium: sodium ratio in the arugula plant compared to the control treatment (Table 5). Also, the potassium: sodium ratio in the arugula plant increased relative to the control treatment by 29%, 53%, 71%, 38%, 89%, 317%, 108%, 113%, 486%, 31%, 96%, and 473% for 1%WCB, 3%WCB, 5%WCB, 1%BLB, 3%BLB, 5%BLB, 1%SSB, 3%SSB, 5%SSB, 1%RSB, 3%RSB, and 5%RSB, respectively. The effectiveness of the treatments on potassium: sodium ratio in arugula plant increase was in the order of 5%SSB > 5%RSB > 5%BLB > 3%SSB > 1%SSB > 3%RSB > 3%BLB > 5%WCB > 3%WCB > 1%BLB > 1%RSB > 1%WCB > control. The lowest potassium: sodium ratio in the arugula plant was observed in the control treatment, while the highest potassium: sodium ratio in the arugula plant was noticed after applying 5%SSB treatment (Table 5).

### Correlations between Biochar dose and plant growth parameters of arugula plant

**Fig. 6:**
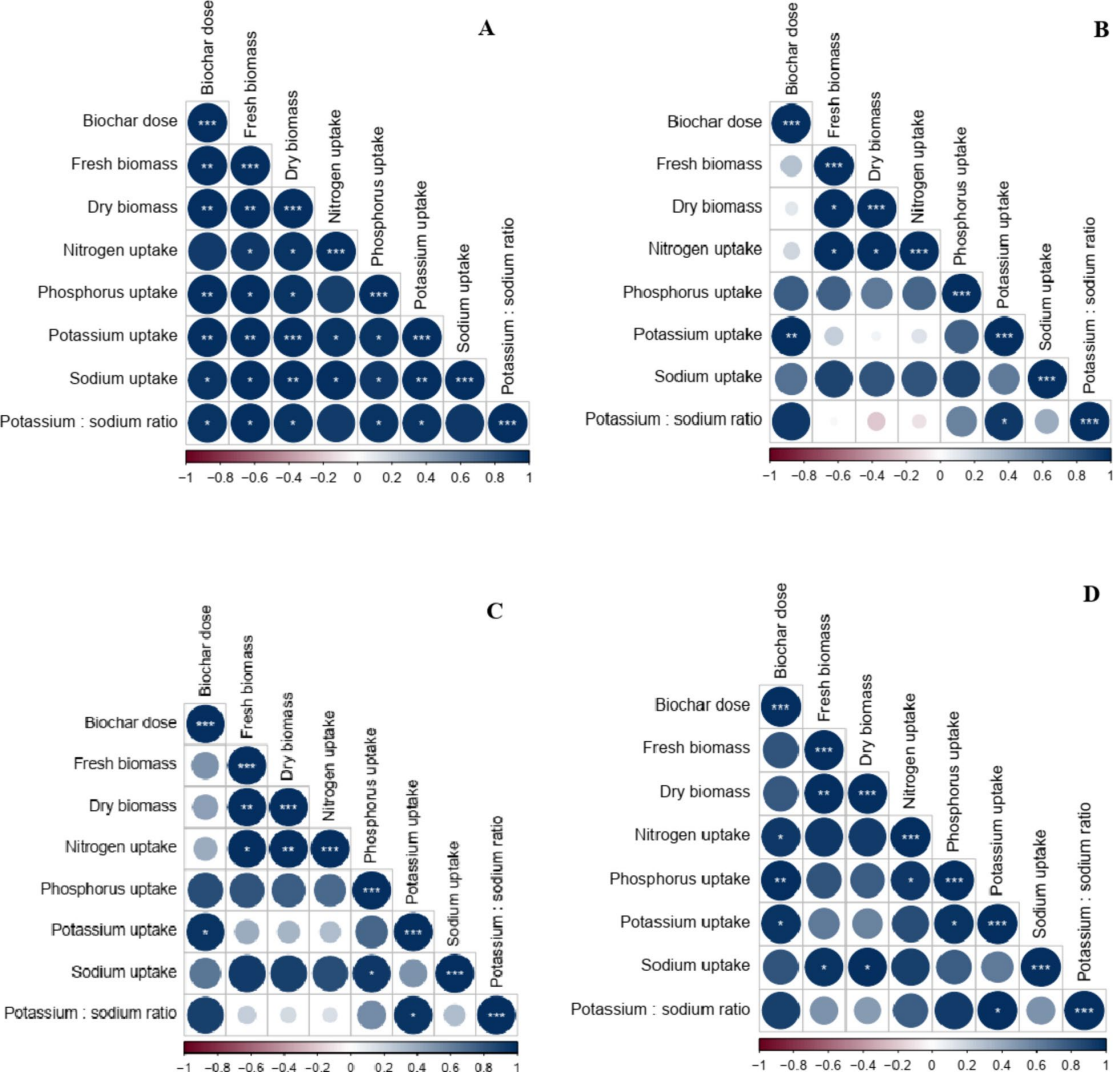
Pearson correlation analysis between biochar dose and plant growth parameters grown in saline sandy with (A) wood chips biochar, (B) banana leaves biochar, (C) sorghum stalks biochar, and (D) rice straw biochar. In the correlation analysis diagram, blue represented a positive correlation; and red represented a negative correlation. ***Correlation is significant at *P* ≤ 0.001; **Correlation is significant at *P* ≤ 0.01; *correlation is significant at *P* ≤ 0.05.

Under applying wood chips biochar in saline soil, the correlation coefficients between biochar dose and fresh biomass, dry biomass, phosphorus uptake, and potassium uptake were positively and highly significant. Biochar dose was positively and significantly correlated with sodium uptake and potassium: sodium ratio. However, the correlation between biochar dose and nitrogen uptake was non-significant positive (Fig. 6A). Biochar dose was highly significant and positively correlated with potassium uptake under banana leaves biochar applications. Moreover, the correlation between the biochar dose and fresh biomass, dry biomass, phosphorus uptake, sodium uptake, and potassium: sodium ratio was positive and non-significant (Fig. 6B). In the presence of sorghum stalks biochar, biochar dose was significant and positively correlated with potassium uptake. Meanwhile, the correlation coefficients between biochar dose and fresh biomass, dry biomass, nitrogen uptake, phosphorus uptake, sodium uptake, and potassium: sodium ratio were non-significant positive (Fig. 6C). Under the application of rice straw biochar, the correlation coefficient between biochar dose and phosphorus was positive and highly significant. Biochar dose was positively significantly correlated with nitrogen uptake and potassium uptake. However, biochar dose and fresh biomass, dry biomass, sodium uptake, and potassium: sodium ratio had a non-significant positive correlation (Fig. 6D).

## Discussion

The type of biomass strongly influenced the physical and chemical properties of biochar^42^. The responses of soil and plants to biochar applications depend on many variables such as feedstock type and pyrolysis temperature, application rate and method of application, and application context (crop, soil type, environmental, and biological stresses)^43^. Our results obtained from this study are compatible with Alotaibi and Schoenau^44^ who reported that amending calcareous sandy soils with biochar increased EC, which is attributed to the presence of soluble salts in the biochar and the adsorbed cations on biochar surfaces which have weak bound by electrostatic forces and dissolve as soluble salts^45^. There is another reason for increasing electrical conductivity in the soil, which is attributed to increasing water-holding capacity and its salts under biochar application^10^. The results obtained from this study were consistent with numerous researchers who found that the electrical conductivity in sandy soil increased with increasing biochar doses under irrigation with saline water^10,46^. Applying biochar to sandy soil significantly increased soluble cations such as Ca, Mg, Na, and K, and their significant increase occurred with increasing the biochar application doses^47^. These increases in soluble cations are attributed to biochar’s high content of alkali and alkaline earth cations^45^. Amending sandy soil with biochar caused a significant increase in the soluble anions such as Cl^−^, HCO_3_^−^, and SO_4_^2−^ and they significantly increased with increasing the doses of biochar application^48^.

In the present study, biochar application to saline soil increased organic matter content, this result was consistent with many studies which found that biochar applications to the soil increased organic matter content^44,49^. Increasing the soil organic matter content improves crop productivity due to increasing soil porosity, improving available nutrients in the soil, and decreasing bulk density^14^. Surface properties of biochar^50^, feedstock type, and biochar application doses were the main factors that significantly affected the CEC in several soils^51,52^. Our results revealed that applying different biochar types increased CEC in saline soil. This finding is supported by previous research showing that amending various soils with many types of biochar resulted in increasing values of CEC^12,49^, this is attributable to the presence of acidic functional groups on biochar surfaces that oxidize, which in turn causes an increase in negative charges^53^ and high surface area of biochar particles^54^. Moreover, CEC is highly dependent on pH and vast differences in surface properties among the different types of biochar^50^. Our results showed that biochar prepared from agricultural residues, such as rice straw, banana leaves, and sorghum straw, has a higher CEC than the biochar produced from wood chips. This result is similar to Kabir et al.^55^, who found that biochar prepared from agricultural residues, such as rice husks and corncobs, has a higher CEC than biochar prepared from wood. Based on the CEC results, the CEC of biochar was mainly affected by the feedstock type, but a little by the environmental conditions^56^. The results obtained from this study agree with many studies, finding that the values of CEC in the soils increased with increasing doses of applied biochar^12,57^. Increasing the CEC in the soil increases its ability to retain cations, which leads to the use of nutrients in the soil and reduces their loss^14^.

Biochar can affect soil nutrients through several mechanisms: firstly, it is a source of nutrients for plants and soil microorganisms, secondly, it retains nutrients, thus influencing nutrient leaching and bioavailability, and thirdly as a soil amendment, it works to change the soil properties, which in turn affects the nutrient cycle and their interactions^58^. Our results were compatible with Amin^10^ who reported that adding biochar to the saline soil resulted in a significant increase in concentrations of available nitrate nitrogen and total available nitrogen, as well as applying biochar in saline soil resulted in a non-significant increase in available ammonium nitrogen. The results obtained from this study were consistent with Wang et al.^59^ who found that the available nitrate in saline-alkali soil increased with increasing biochar doses. The results obtained from this study are supported by previous research suggesting that the concentration of available nitrate was higher than ammonium concentrations in the soils amended with biochar is attributed to the increasing activity of the nitrification process^60^. Co-application of biochar with nitrogen fertilizers increased nitrate retention in the soils due to increasing water holding capacity^61,62^. Generally, factors affecting available nitrogen in the soil after biochar application include residence time of biochar in soil, fertilization, soil properties, biochar application dose, feedstock type, and CEC^63^. Our study demonstrated that biochar applications to saline soil improved phosphorus availability, these findings were consistent with many studies suggesting that applying biochar to the soils increased phosphorus availability and is attributed to releasing phosphorus from biochar, which has a large amount of phosphorus^31,64^. In this study, the availability of phosphorus and potassium increased with the different types of biochar and their increasing doses, a finding that aligns with Amin^12^, who found that the availability of phosphorus and potassium enhanced significantly in calcareous sandy soil with different types of biochar and also increased with increasing biochar levels. Applying biochar types to this soil showed increases in available phosphorus in the order of sorghum panicle biochar > orange peel biochar > wood chips biochar^12^. Our study has illustrated that available phosphorus increases ordered as follows: BLB > SSB > RSB > WCB. However, the application of biochar types in this soil showed increases in available potassium in the order of orange peel biochar > sorghum panicle biochar > wood chips biochar^12^. However, our results revealed the increase in soil available potassium in the order of RSB > BLB > SSB > WCB. Adding biochar to saline soil may increase the potassium concentration in the soil, depending on the raw materials. Increases of available potassium in biochar-amended saline soil are because of biochar richness in potassium itself and its porous structure provides an ideal environment for potassium-solubilizing microorganisms^65^. Moreover, biochar application at high doses caused an increase in the nutrient-holding capacity of sandy soils, which in turn leads to preventing their leaching and making them available to plants^66^.

The results showed that adding all biochar treatments to saline soil did not significantly affect the chlorophyll of the arugula plant. This finding is supported by previous research showing that the chlorophyll fluorescence parameters of stressed arugula plants grown under salinity conditions did not differ significantly^29^. The ability to understand the interaction between abiotic stresses and soil amendments has high economic importance^67^. Using saline water to produce vegetables is one of the main challenges for producers because salt stress causes a decline in crop production and yield^68^. Our experiment showed that applying all types of the added biochar resulted in a significant increase in fresh and dry biomass of arugula plant grown in saline soil, which agrees with Zhou et al.^69^ asserting that the shoot and root biomass of arugula plants were enhanced significantly under biochar addition to the soil. Increasing salinity significantly affected the sodium content in all arugula plants; increasing sodium concentration in the roots and shoots happened with increasing salinity levels. Cations balance such as potassium, calcium, and magnesium play a vital role in varying the effects of salinity resulting from increasing sodium concentration^70^. The results obtained from this study revealed that applying different types of added biochar mitigates the salinity stress and enhances arugula growth, which agrees with other results in adding biochar to salt-affected soils can alleviate salinity-induced stress and directly enhance the growth of plants. Applying almond shell biochar to saline soil at a level of 5% led to the highest fresh and dry biomass of arugula plants^71^. Amending soil with phosphoric acid-modified biochar significantly improved the fresh and dry biomass and phosphorus content of the arugula plant^72^. Adding nitrogen fertilization to the soil improves growth parameters and production of arugula^73^. On the other hand, the growth parameter of the arugula plant grown in calcareous sandy loam soil was negatively affected by saline irrigation. This is due to the impact of plant physiological processes resulting from increased salinity in the soil such as reducing photosynthetic rate, altering stomatal conductance, and nutritional imbalance^70^. Biochar efficiency in improving crop productivity and soil salinity in salt-affected soils is highly dependent on biochar properties, soil properties, climatic conditions, in addition to agricultural and management practices. Moreover, the key mechanisms contributing to the positive response of crop productivity also changed for the same biochar under different climatic conditions, soil properties as well as agricultural and management practices^74^. Also, it is believed that biochar’s effect on plant productivity in salt-affected soil depends on the feedstock type, pH, and application doses of biochar^16^. Biochar application as an amendment is considered a promising strategy for improving crop productivity in salt-affected soils, alleviating the negative impacts of salt stress in crops in the season^75,76^. Several mechanisms have been proposed for reducing salinity stress in soils amended with biochar including (1) release of essential macro- and micronutrients, such as calcium, potassium, nitrogen, phosphorus, and zinc into the soil, as these nutrients play a vital role in counteracting the negative consequences of high salinity concentrations^20^, (2) increasing Na^+^ adsorption, (3) increasing soil water holding capacity with biochar addition may cause dilution of salt in soil solution which in turn reduces osmotic stress, (4) increasing availability of many nutrients in soil solution led to decreasing Na^+^ uptake causing a reduction in salinity stress in plants^75,76^, (5) raising the potassium: sodium ratio within the plants appears to be a vital strategy that increases the salt tolerance of plants and subsequently promotes plant growth and enhances yield, thus highlighting a great benefit correlated with using biochar as an amendment in salt-affected soils^20,74^, (6) increasing photosynthetic indices and activity of antioxidant enzymes, illustrating that biochar plays a vital role in alleviating the salt stress in the soil^21^, (7) decreasing oxidative stress under salinity stress, this is attributed to the fact that the application of biochar regulates the synthesis of antioxidant enzymes in plants, thus reducing salt stress in plants^77^. In the present study, nitrogen and phosphorus content in arugula plant increased significantly with applying biochar to the soil. This finding is consistent with the previous report, in which incorporating biochar with the soil significantly improved nitrogen and phosphorus concentrations in the root and shoot of radish plants^78^. This study demonstrated that applying biochar significantly increased the potassium: sodium ratio in arugula plant grown in saline soil because of increasing available potassium in salt-affected soils. Applying biochar increases silicon in the soil thereby improving crop yield^14^ this is attributed to silicon’s ability to mitigate salinity stress in plants because it often reduces sodium ions in the shoot of plants and increases the ratio of potassium: sodium^79^. Also, adding silicon nanoparticle-based biochar to the soil under salinity stress enhances the physicochemical properties of the soil as well as improves the physiology and growth of wheat. This may be attributed to promoted plant growth, increased water-holding capacity, increased nutrient supply, and increased stress tolerance^80^.

The study’s limitations are conducting field experiments to select the appropriate type and doses of biochar and assess their long-term effects on soil properties and productivity of several crops under saline conditions as well as performing economic analysis and commercial viability of production and application of biochar to the soils in sustainable agricultural production. These study’s gaps illustrate the current limitations and constraints in comprehending the use of different types and doses of biochar under saline conditions. Moreover, they also give chances for future research and innovative solutions for sustainable agricultural practices.

## Conclusion

The problems of soil salinization and freshwater shortages represent one of the biggest challenges facing food production worldwide to ensure food security. Therefore, managing and using saline water and saline soil in agriculture to produce food crops is considered one of the goals of agricultural development to achieve food self-sufficiency. The type and doses of biochar applied to saline soils play a vital role in plant tolerance to salt stress. In this study, adding biochar to soil helped alleviate nutrient deficiency and crop failure under salt stress conditions. The addition of biochar to saline soil improved nutrient availability and cation exchange capacity. The fresh biomass of the arugula plant increased significantly with the application of different types of biochar under saline conditions. The results indicated that applying sorghum stalks biochar at a dose of 3% effectively enhanced arugula plant growth under salt stress. This is due to the enhancement of nutrient uptake, productivity, and growth of arugula plant under saline conditions as it improves the plant’s tolerance to salt stress as well as improving soil quality. Also, adding 3% sorghum stalks biochar saves the costs of addition and production compared to adding a 5% dose. Based on the results obtained from this study, it is preferable to apply sorghum stalks biochar at a dose of 3%. Therefore, biochar applications to soil may be a promising strategy for improving crop productivity under salinity stress. This study provided useful information on the amounts and types of biochar used to improve soil properties and crop productivity in saline soils. The use of marginal soils, which are usually low-fertile and affected in varying degrees by stresses of nutrients, salinity, and others, to produce alternative products without a significant reduction in the economic value of the main product; seems to be a practical solution and a promising strategy in sustainable agricultural development. In addition, biochar can also contribute to promoting a circular economy approach by recycling agricultural residues through its use in agriculture and enhancing environmental sustainability. Future vision: It is recommended that more research and field experiments be conducted to choose appropriate doses of biochar applications and evaluate their long-term effects on soil quality indicators and crop productivity in saline soils under irrigation with saline water. Moreover, future studies will be performed to investigate the effects of biochar in mitigating abiotic stresses such as salinity and drought on plants and study the effect of biochar properties such as particle size and pyrolysis temperature on its effectiveness under abiotic stresses.

## Data Availability

The datasets used or analyzed during the current study are available from the corresponding author upon reasonable request.
